# Living in the city: *Angiostrongylus cantonensis* is a novel threat to an urban population of Florida burrowing owls (*Athene cunicularia floridana*) in south Florida

**DOI:** 10.1186/s13071-025-06700-7

**Published:** 2025-02-26

**Authors:** Håkon Jones, Nicole M. Nemeth, Brittany Piersma, Rebecca Hardman, Lisa A. Shender, Raoul K. Boughton, Kayla B. Garrett, Nikole Castleberry, P. J. Deitschel, Xuan Hui Teo, Rebecca Radisic, Martha Frances Dalton, Michael J. Yabsley

**Affiliations:** 1 Southeastern Cooperative Wildlife Disease Study, College of Veterinary Medicine, University of Georgia, Athens, GA 30602 USA; 2 Department of Pathology, College of Veterinary Medicine, University of Georgia, Athens, GA 30602 USA; 3 Audubon Western Everglades, 12250 Tamiami Trail E. Suite 309, Naples, FL 34113 USA; 4 Florida Fish and Wildlife Conservation Commission, Fish and Wildlife Research Institute, St. Petersburg, FL 33701 USA; 5 Florida Fish and Wildlife Conservation Commission, 1105 SW Williston Rd, Gainesville, FL 32601 USA; 6 National Park Service, Biological Resources Division, Wildlife Health Branch, Fort Collins, CO USA; 7 The Mosaic Company, 414 W Main St, Wauchula, FL 33873 USA; 8 Warnell School of Forestry and Natural Resources, University of Georgia, Athens, GA 30602 USA; 9 Conservancy of Southwest Florida, 1495 Smith Preserve Way, Naples, FL 34102 USA; 10 Center for Ecology of Infectious Diseases, University of Georgia, Athens, GA 30602 USA; 11 Wildlife Health Building, Southeastern Cooperative Wildlife Disease Study, Department of Population Health, University of Georgia, 589 D.W. Brooks Drive, Athens, GA 30602 USA

**Keywords:** Rat lungworm, *Angiostrongylus cantonensis*, Burrowing owl, Neurologic, Parasite, *Athene cunicularia*, Wildlife health, Florida

## Abstract

**Background:**

*Angiostrongylus cantonensis*, the rat lungworm, is a metastrongyloid parasite that uses rodents as definitive hosts, mollusks as intermediate hosts, and a wide range of invertebrate and vertebrate species as paratenic hosts. Although this parasite poses a significant public health concern in many regions of the world, it can also cause disease in numerous domestic and wildlife aberrant host species. When parasite larvae are ingested by one of these aberrant hosts, larval migration in the central nervous system causes extensive damage, resulting in spinal cord and/or brain damage and inflammation, leading to potentially fatal neurological disease. We describe *A. cantonensis* infection in a novel host, the Florida burrowing owl (*Athene cunicularia floridana*), on Marco Island, Collier County, Florida, USA. The Florida burrowing owl is a state-listed species that has experienced steep population declines across its range, primarily due to habitat loss and fragmentation. Many populations are now restricted to urban environments, which pose novel threats to the owls, such as exposure to anticoagulant rodenticides and novel pathogens, increased risk of predation, vehicular strike, and increased disturbance at nest sites.

**Methods:**

Through diagnostic evaluation of carcasses and select tissues submitted to the Southeastern Cooperative Wildlife Disease Study from 2019 to 2023, we diagnosed nine confirmed or suspected cases of angiostrongylosis on Marco Island.

**Results:**

Microscopic examination and polymerase chain reaction (PCR) testing confirmed parasite identification. In addition, ancillary testing ruled out other potential causes of neurological disease, such as rodenticides, West Nile virus, and highly pathogenic avian influenza virus.

**Conclusions:**

This study underscores the importance of surveillance and monitoring efforts for *A. cantonensis*, particularly in regions where novel hosts may serve as indicators of public health risk. In addition, as urbanization and habitat fragmentation continue encroaching upon wildlife habitats, understanding the dynamics of host–parasite interactions becomes crucial for mitigating the spread of zoonotic diseases.

**Graphical Abstract:**

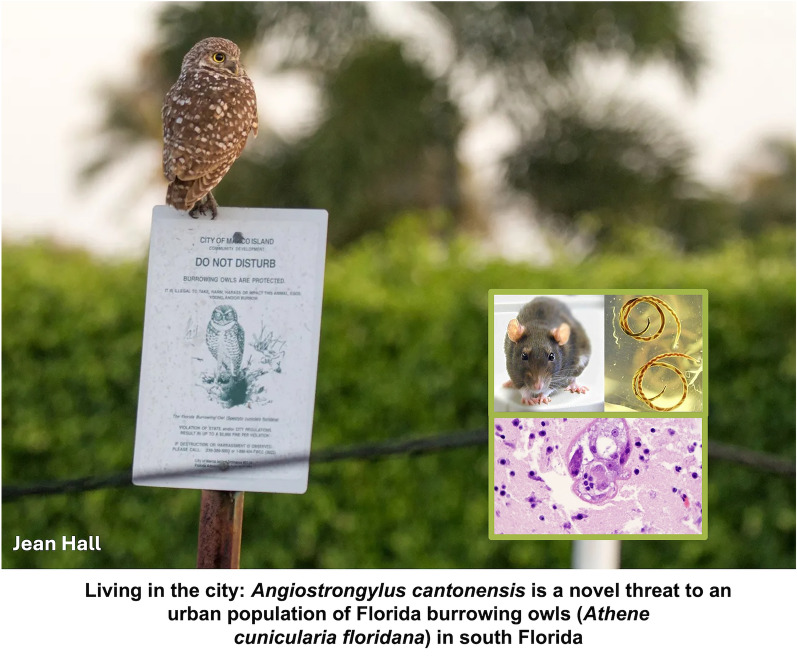

## Background

The rat lungworm, *Angiostrongylus cantonensis*, is a metastrongyloid parasite that uses rodents, primarily those in the genus *Rattus*, as definitive hosts, and a high diversity of mollusks as intermediate hosts [1–8]. This parasite can also use many invertebrate (e.g., crustaceans and insects) and vertebrate species (e.g., frogs and lizards) as paratenic hosts [9–14]. When a definitive host ingests infective *A. cantonensis* larvae, they migrate to the central nervous system (CNS) where they undergo two molts into young adults. In natural definitive hosts, they then migrate to the pulmonary arteries where they mature, mate, and release L1 larvae that migrate to the pharynx and are passed in the feces. However, when ingested by an aberrant host, the larval migration in the CNS causes extensive damage, resulting in spinal cord and/or brain damage and inflammation, leading to potentially fatal neurological disease, that may include ataxia, head tremors, circling, stupor, and paralysis [3, 7, 15–17]. 

This parasite is an important cause of disease in humans, and also in numerous aberrant domestic and wild mammalian and avian host species [1, 3]. Because *A. cantonensis* has a broad geographic distribution, this parasite is now recognized as a leading cause of eosinophilic meningitis worldwide, with thousands of documented human cases in 30 countries and major outbreaks occurring in China, Taiwan, and Thailand [1, 4, 18]. In the USA, most cases to date have been documented in Hawaii; however, sporadic autochthonous human cases have been reported from the southern continental USA, including three recent cases in Florida [1, 6, 19, 20]. In addition to being a threat to public health, this parasite is an important pathogen of dogs, captive exotic species, and certain wildlife species, such as tawny frogmouths (*Podargus strigoides*) and brushtail opossums (*Trichosurus vulpecula*) [21]. Because of high human case numbers in some regions, animal cases have been suggested as sentinels for angiostrongyliasis public health risk [1, 7, 22].

Although *A. cantonensis* is endemic to Asia, infected rats have facilitated the spread of this parasite to the USA and many other regions of the world [2, 23, 24]. In the USA, infections were first documented in brown rats (*Rattus norvegicus*) in New Orleans, Louisiana, in 1987, likely originating from rats coming ashore from incoming ships. Early reports of infections in mammalian incidental hosts included a horse, a wild woodrat (*Neotoma floridanus*), wild armadillos (*Dasypus novemcinctus*), and wild opossums (*Didelphis virginiana*) from Louisiana, Mississippi, and Florida [25, 26]. In addition, infections in captive nonhuman primates and lemurs have been reported in Texas, Louisiana, Florida, and Alabama [15, 27–32]. Because of the high diversity of potential gastropod intermediate hosts, paratenic hosts, and widespread distribution of multiple definitive host rodent species, it is believed that *A. cantonensis* is, or will soon be, endemic across a wider range of the USA than currently is recognized [5, 11, 23, 29, 33–36]. In fact, a novel rat host (*Sigmodon hispidus*) was recently confirmed in Oklahoma [36].

The burrowing owl (*Athene cunicularia*) is a small, long-legged owl that lives in different types of open landscapes throughout North and South America (i.e., grasslands, rangelands, agricultural areas, and deserts) [37, 38]. These owls are active during the day and nest and roost in burrows excavated by themselves or by reptiles and mammals [37, 38]. There are approximately 18 recognized subspecies, 1 of which, the Florida burrowing owl (*A. c. floridana*), occurs in Florida, and possibly the Caribbean. This non-migratory subspecies is classified as State Threatened by the Florida Fish and Wildlife Conservation Commission [38]. Historically, these owls occupied prairies throughout Florida north of the Everglades, but owing to habitat loss, the Florida burrowing owl now has a highly patchy distribution, with some populations occurring in residential lawns and vacant lots in suburban environments. The human-driven increased use of developed areas has increased the diversity of threats faced by owls, including predation by domestic animals, anticoagulant rodenticide toxicity, vehicular collisions, harassment, and infectious and parasitic diseases [38–40]. Herein, we provide details, including molecular characterization, on several suspected and confirmed cases of *A. cantonensis* diagnosed from 2019 to 2023, in a novel host; the Florida burrowing owl.

## Methods

The cases of angiostrongylosis reported consist of burrowing owl carcasses and field-collected fresh and formalin-fixed tissue sets submitted to the Southeastern Cooperative Wildlife Disease Study Research and Diagnostic Service at the University of Georgia (Athens, GA) for diagnostic evaluation (Table 1). Standard procedures were followed for the gross examination of all organs, collection, and preservation for histopathologic examination, and ancillary testing appropriate for clinical history and postmortem findings. Tissue samples were collected and fixed in 10% neutral buffered formalin. The brain, heart, lung, trachea, liver, kidney, spleen, skeletal muscle, adrenal gland, spleen, proventriculus, ventriculus, and small and large intestine were collected from all owls. If lesions were grossly evident (or if opportunistically trimmed in with other organs), the following organs were sampled: crop, eye, spinal cord, nasal sinuses, cloacal bursa, and adipose (if present). Fixed tissue samples were routinely processed, embedded in paraffin wax, sectioned at 5 µm, and stained with hematoxylin and eosin (H&E). In cases for which no parasites were histologically evident, additional 5-µm step cuts were done on brain sections for further evaluation. Fresh samples of brain, lung, liver, spleen, kidney, and small intestine were also collected at necropsy and frozen at −20 °C. Gross and histologic evaluation was performed by a board-certified anatomic veterinary pathologist.

**Table 1:** Demographic and diagnostic data from nine Florida burrowing owls (*Athene cunicularia floridana*) in Collier County, Florida, USA, that were suspected (3) or confirmed (6) to have *Angiostrongylus cantonensis*-associated disease

Case	Status^a^	Date signs noted/date of death	Sex/age	Weight (g)	*Angiostrongylus* diagnostics			Notes
					Brain squash	Histology	PCR results	
1	Suspect	3 March 2019/20 March 2019	Male juvenile	115.0	Negative	Negative	Negative	
2	Confirmed	10 April 2019/10 April 2019	Female juvenile	100.0	Negative	Positive	Positive (66-kD)	
3	Suspect	19 April 2020/19 April 2020	Female juvenile	126.0	Negative	Negative	Negative	Sibling of W20-374B
4	Confirmed	14 May 2020/14 May 2020	Unknown juvenile	106.5	Negative	Positive	Positive (66-kDa)	Sibling of W20-374A
5	Confirmed	7 April 2020/7 April 2020	Male juvenile	123.0	Negative	Positive	Positive (66-kDa)	
6	Confirmed	19 April 2021/19 April 2021	Male fledgling	139.7	Negative	Negative	Positive (18S rRNA)	Sibling of W21-269B
7	Suspect	19 April 2021/20 April 2021	Male fledgling	112.2	Negative	Negative	Negative	Sibling of W21-269A
8	Confirmed	26 May 2023/1 June 2023	Female juvenile	113.5	Positive	Positive	Positive (66-kDa)	
9	Confirmed	30 June 2023/30 June 2023	Male juvenile	117.1	Negative	Negative	Positive (66-kDa)	

^a^Confirmation was via molecular identification of *A. cantonensis* in a fresh brain sample and nematodes were detected in brain squashes and via histology of several owls

Nematodes were extracted from the brain and viewed under dissecting and compound microscopes for morphological identification when possible [41–43]. DNA was extracted from the nematodes using a commercial kit (DNeasy, Qiagen, Valencia, California). A portion of the 18S rRNA (950 bp) gene was amplified using primers 18S39F and 18S977R [44]. For the 18S rRNA gene sequence, a phylogenetic tree was constructed using an approximately maximum-likelihood method with FastTree v2.1 with a generalized time-reversible (GTR) model in Geneious Prime 2024.0.7 (Biomatters Limited, Auckland, New Zealand). *Metastrongylus salmi* was used as an outgroup and for rooting the tree, as has been done previously [45, 46].

For owls in which brain samples had no visible nematodes, or if brains were not examined grossly, DNA was extracted from brain samples and tested for *A. cantonensis* using primers AC1 and AC2 that target the 66-kDa protein gene [47]. Amplicons were gel-purified using a gel-purification kit (Qiagen) and bi-directionally sequenced at Genewiz (Plainfield, New Jersey). Chromatograms were analyzed using Geneious (Auckland, New Zealand), and the consensus sequence was compared with other sequences in the GenBank database.

Additional diagnostic evaluation and testing was conducted on these animals that aimed to determine the cause of neurologic disease; thus, the brain was a specific focus. Brain samples underwent virus isolation targeting select avian arthropod-borne viruses (e.g., West Nile virus and eastern equine encephalitis virus) [48] and *Toxoplasma gondii* and other apicomplexan parasites by PCR, using primers Tg18s58F and Tg18s348R, as described previously [49]. Beginning in 2023, pooled oropharyngeal and cloacal swabs from each owl were tested for avian influenza virus. Nucleic acids from pooled swabs were extracted using the MagMAX96 AI/ND Viral RNA isolation kit (Ambion/Applied Biosystems, Foster City, CA) [50] and screened using a real-time reverse transcription-PCR (rRT-PCR) assay specific for Gs/GS 2344b H5 [51].

## Results

**Fig. 1:**
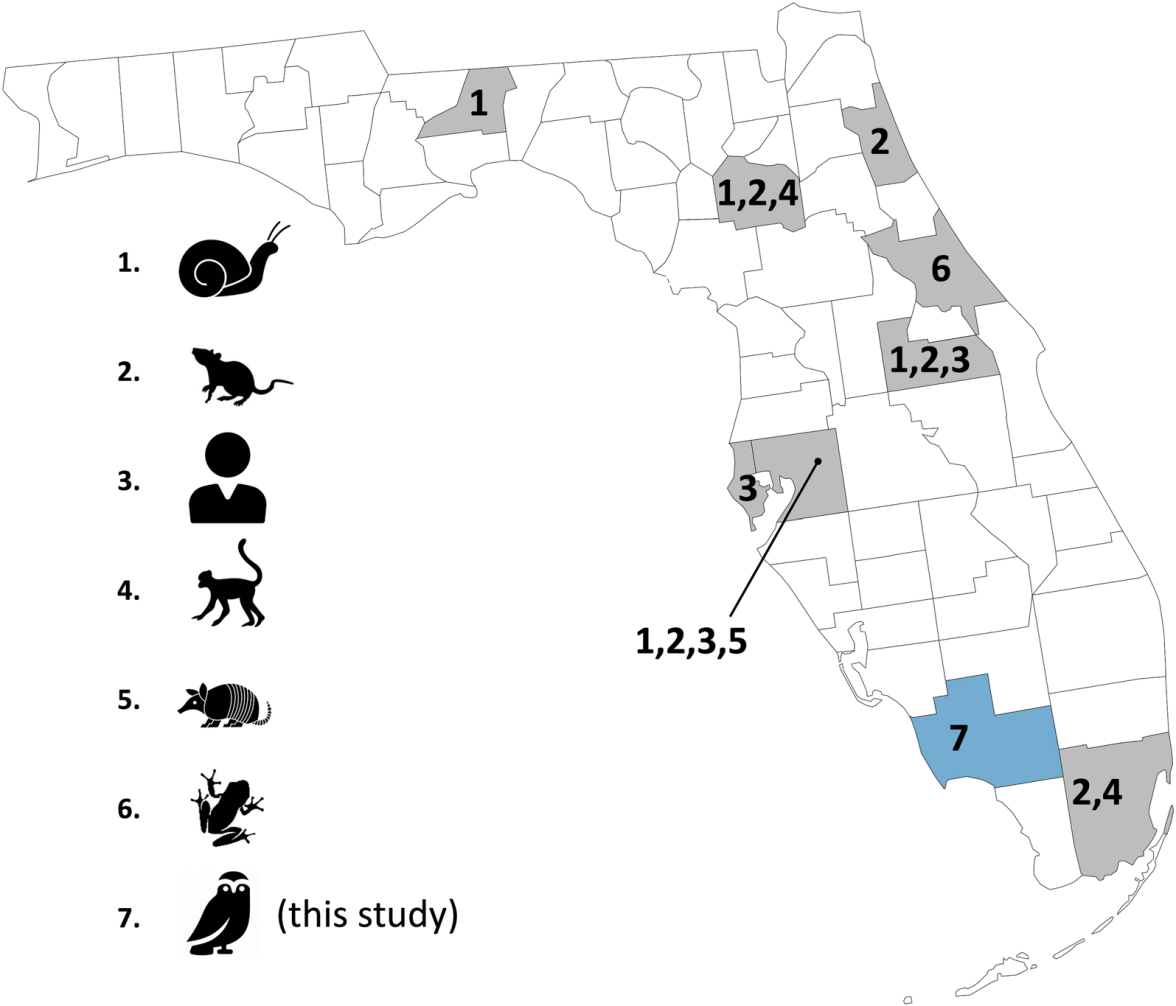
Distribution and mollusk and/or vertebrate host range for *Angiostrongylus cantonensis* in Florida, USA. Burrowing owls (*Athene cunicularia*) from the current study are shown in blue (number 7). Positive mollusks (number 1) included numerous species. All positive rats (number 2) were black rats, *Rattus rattus*. Multiple species of nonhuman primates (number 4) have been reported. number 5 represents a single positive nine-banded armadillo (*Dasypus novemcinctus*), and number 6 represents a positive Cuban tree frog (*Osteopilus septentrionalis*). Previously published reports included: [10, 20, 26, 29, 31, 32, 35]

For several years prior to 2019, regional biologists reported occasional sightings of burrowing owl nestlings with neurologic signs in the same area. Since 2020, six fatally infected burrowing owls were diagnosed postmortem with *A. cantonensis*-associated encephalitis, which was confirmed microscopically by morphology of nematodes and/or PCR testing. An additional three owls from 2019 and 2021 had clinical signs and histologic lesions that were highly suggestive of aberrant nematode migration, and were considered suspect cases of *A. cantonensis*. All nine cases were from Marco Island, Collier County, Florida, and were submitted during the nesting season from March to June (Fig. 1).

### Case 1 (W19-521/CC19-133)

This burrowing owl nestling was observed to have neurologic signs of tremors and incoordination on 3 March 2019, and its mobility was severely impaired. Of its two siblings, one remained neurologically normal and the other displayed mild ataxia that appeared to resolve within a week of observation. The affected nestling was brought into captivity for rehabilitation on 4 March 2019, where incoordination, head tilt, horizontal head tic, and hind limb extensor rigidity were noted. During treatment, it was alert, vocalizing, and responded to hand feeding. However, owing to lack of improvement, it was euthanized using isoflurane gas inhalation 16 days after admission (20 March 2019), and the carcass was refrigerated until it was shipped to SCWDS the following day for necropsy.

**Fig. 2:**
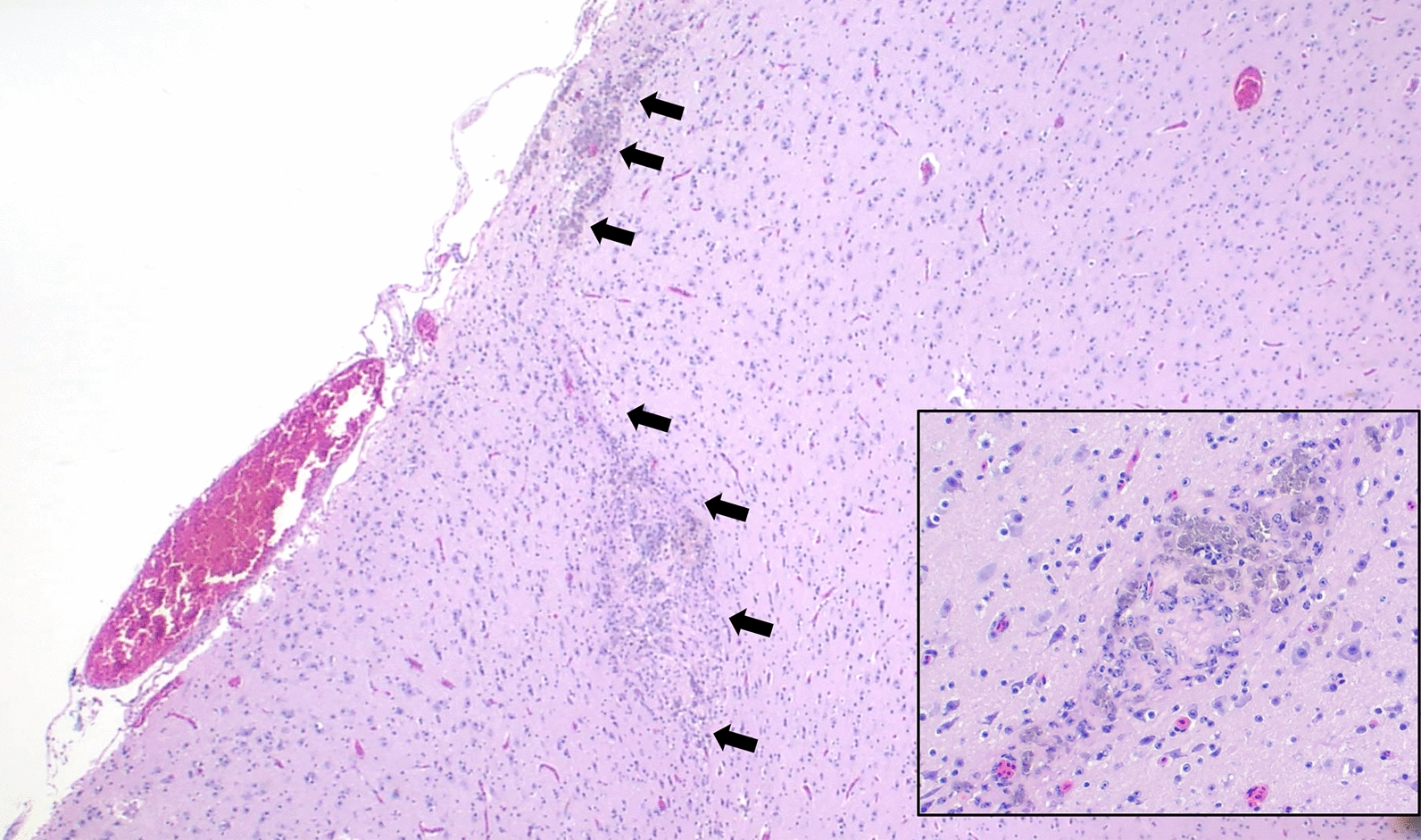
Suspected *A. cantonensis* infection in a burrowing owl (*Athene cunicularia*) in Florida. W19-521/CC19-133 (2× H&E). Irregular (presumed nematode migration) track (arrows) in the superficial cerebrum that disrupts the neuropil, causing rarefaction and infiltration by abundant hemosiderophages (inset; 10×), microglial cells including gitter cells, and less commonly, multinucleated giant cells

The owl was in fair nutritional condition with minimal postmortem autolysis. The blood vessels over the cerebrum and cerebellum were congested. Microscopically, the neuroparenchyma was multifocally disrupted by widespread, irregularly linear tracks of rarefied neuroparenchyma infiltrated by abundant hemosiderophages, microglial cells including gitter cells, and less commonly, multinucleated giant cells (Fig. 2). Occasionally the tracks extended to ventricular ependymal borders and numerous sloughed ependymal cells were within corresponding ventricles. Blood vessels within, and immediately adjacent to, the tracks were surrounded by perivascular, lymphocytic cuffs of 1–5 cell layers thick. In the cervical spinal cord, there was mild spongiosis characterized by linear tracks of clear to wispy vacuoles (dilated myelin sheaths) with rare spheroids. Other histologic lesions included mild, multifocal, chronic, lymphohistiocytic, necrotizing perivertebral myositis and moderate, focal, acute, hemorrhage in the intestinal serosal adipose and adrenal gland.

At the time of initial laboratory testing in 2019, a very short nematode sequence was obtained and was thought to support the presumption of *Baylisascaris* neural larval migrans, but a causative agent was not definitively determined. After *A. cantonensis* cases from the island were confirmed over the next several years, DNA was extracted from formalin-fixed paraffin embedded (FFPE) brain tissue and tested for *A. cantonensis*. The PCR result was negative, so this case remains suspect. No viruses nor protozoa were detected in fresh brain samples.

### Case 2 (W19-587/CC19-193)

This chick displayed neurological signs (head tremors and lethargy) when it was live trapped, along with the parents and three siblings, in April 2019. The owl chick gradually became obtunded and died later that day. The carcass was frozen and submitted to SCWDS for postmortem evaluation.

The juvenile owl was in poor nutritional condition, and postmortem autolysis was mild. There was mild urate staining around the vent. At necropsy, the meningeal blood vessels over the right hemisphere were congested, and there was focal hemorrhage within the right lung lobe, with a 1 mm depression in the superficial parenchyma.

Histologically, there was minimal perivascular, lymphocytic cuffing in the cerebrum with few hemosiderophages, moderate neuroparenchymal rarefaction, and a degenerated nematode in the adjacent neuroparenchyma. The nematode was 50–75 μm in diameter, lacked lateral alae, had coelomyarian musculature, and a gastrointestinal tract lined by multinucleated cells. There was multifocal, mild, acute superficial- to mid-myocardial hemorrhage and moderate, focal, pulmonary hemorrhage.

Because *Baylisascaris* larval migrans were suspected as the cause of the brain lesions, multiple fresh brain samples were squashed and examined for nematode larvae, but none were detected. Retroactive PCR testing of FFPE brain tissue using the 66-kDa gene was positive and the sequence was 100% similar to numerous *A. cantonensis* sequences in GenBank. No viruses nor protozoa were detected in fresh brain samples.

### Cases 3 and 4 (W20-374A and B)

Two juvenile burrowing owl siblings exhibited neurologic signs, including apparent visual impairment (unresponsive to visual stimuli), dilated pupils, and inability to stand; they exhibited these signs approximately 1 month apart (the first, owl A, on 19 April 2020 and the second, owl B, on 14 May 2020). These owls were admitted to a wildlife rehabilitation clinic, where they were euthanized using isoflurane gas inhalation, and the carcasses were frozen prior to submission for necropsy. At necropsy, owl A had crusty debris around both nares, and the oral cavity contained stringy mucus. The owl was in good nutritional condition with moderate to abundant fat stores. Owl B had slightly sunken corneas and dry, powdery material in both nares. The choanal slit contained stringy mucus and feathers surrounding the vent were urate stained. Nutritional condition was fair, with moderately prominent keel bone and a moderate amount of internal adipose stores.

**Fig. 3:**
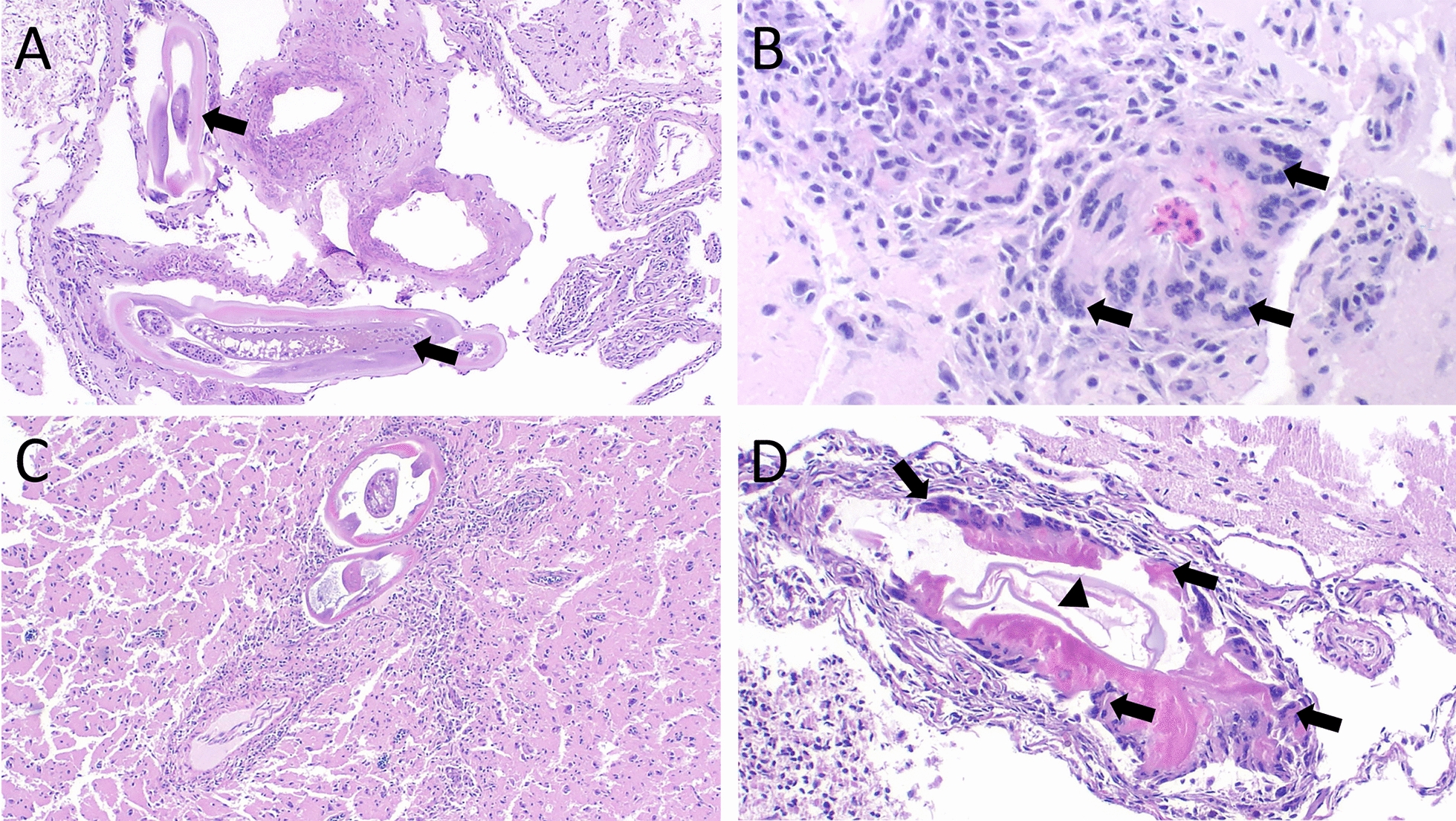
Confirmed *A. cantonensis* infection in two burrowing owls (*Athene cunicularia*) in Florida. **A** W20-374B-4 (4× H&E). Within the meninges overlying the cerebrum, multiple nematode segments (arrows) are surrounded by thick bands of inflammatory cells (i.e., multinucleated giant cells, histocytes, and heterophils) and necrosis, while the surrounding meninges are expanded by moderate numbers of lymphocytes and plasma cells. **B** W20-374C-7 (20× H&E). Adjacent to a nematode (not in view), the cerebrum is focally infiltrated by numerous multinucleated giant cells (arrows) enclosing a small cluster of degenerated heterophils; the surrounding neuroparenchyma is infiltrated by lymphocytes, plasma cells, and pyknotic debris. **C** W23-481-3A (4× H&E). The cerebrum is multifocally disrupted by multiple nematode segments (likely from the same nematode) surrounded by high numbers of lymphocytes, plasma cells, histiocytes, and hemisoderophages. The fragmented tissue in the surrounding normal neuroparenchyma represents a freeze–thaw artifact. **D** W23-481-6A (10× H&E). The meninges over the cerebrum are infiltrated by loose aggregates of lymphocytes and plasma cells with a few scattered hemosiderophages, and an inner, denser band of multinucleated giant cells (arrows) and necrotic debris encircling a degenerated nematode (arrowhead)

Histologic lesions in owl A were limited to mild thickening of the leptomeninges over the cerebrum. In its sibling, owl B, the superficial cerebral parenchyma and overlying meninges had numerous sections of nematodes that were ~100 μm in diameter with a ~5 μm thick, smooth cuticle, coelomyarian–polymyarian musculature, pseudocoelom with prominent lateral chords and alae, and an intestinal tract lined by uninucleated, columnar cells with a brush border. The nematodes were within variably sized foci of necrotic neuroparenchyma surrounded by multinucleated giant cells, histocytes, lymphocytes, and plasma cells (Fig. 3A). Occasionally, degenerated nematode cross sections were surrounded by a band of multinucleated giant cells and loosely scattered inflammatory cells as described above.

Brain samples from both chicks tested PCR negative for *A. cantonensis* and other nematodes; however, the morphology of the nematode observed in the tissue of owl B is consistent with nematodes observed in owls with confirmed *A. cantonensis* infections. No viruses or protozoa were detected in a fresh brain sample from owl B. Owl A was PCR negative for protozoa, but fresh tissues were not available for virus isolation.

### Case 5 (W20-374C)

This juvenile owl was found at the entrance of its burrow on 7 April 2020; it was repeatedly falling over and seemingly unable to stand. It was transported to the Von Arx Hospital where it was euthanized using isoflurane gas inhalation owing to poor prognosis. Histopathology revealed multifocal, mild to moderate, widely scattered eosinophilic and granulomatous meningoencephalitis, with variable numbers of surrounding lymphocytes, plasma cells, and multinucleated giant cells. At least three foci of granulomatous, heterophilic inflammation encompassed areas of necrosis and aggregates of variably degenerated heterophils (Fig. 3B), sometimes surrounding poorly discernible parasite cross sections. A portion of brain tissue was PCR positive for *A. cantonensis.*

### Cases 6 and 7 (W21-269A and B)

These sibling fledglings were from a burrow on Marco Island, Florida. They both exhibited neurologic signs, including dull mentation and lateral recumbency, seemingly unable to stand. The first (owl A) also had hind limb extensor rigidity, while the second (owl B) also lacked response to visual stimuli and had a head tilt to the left with intermittent head tremors. Two adults (presumed parents) at the burrow were reportedly clinically normal at the time. Owls A and B were euthanized using isoflurane gas inhalation on 19 and 20 April 19 2021, respectively. The carcass of fledgling A was stored frozen, while B was submitted fresh for necropsy.

Both fledglings were in fair to good nutritional condition, with moderate visceral fat stores and pectoral musculature robustness, and minimal autolysis. Owl A had moderate hemorrhage along the inside of the left temporal and frontal (skull) bones that extended to the left ocular globe. The mid to caudal lung lobes were bilaterally dark red and firm (more severe in the left than right). Owl B had vascular congestion and suspect hemorrhage in the left ventral cerebrum.

Histologically, owl A lacked significant lesions (but with only a small section of brain available for examination). Owl B had multifocal, mild, perivascular histiocytic and lymphoplasmacytic encephalitis with rare pyknotic debris within a region of the cerebral gray matter.

**Fig. 4:**
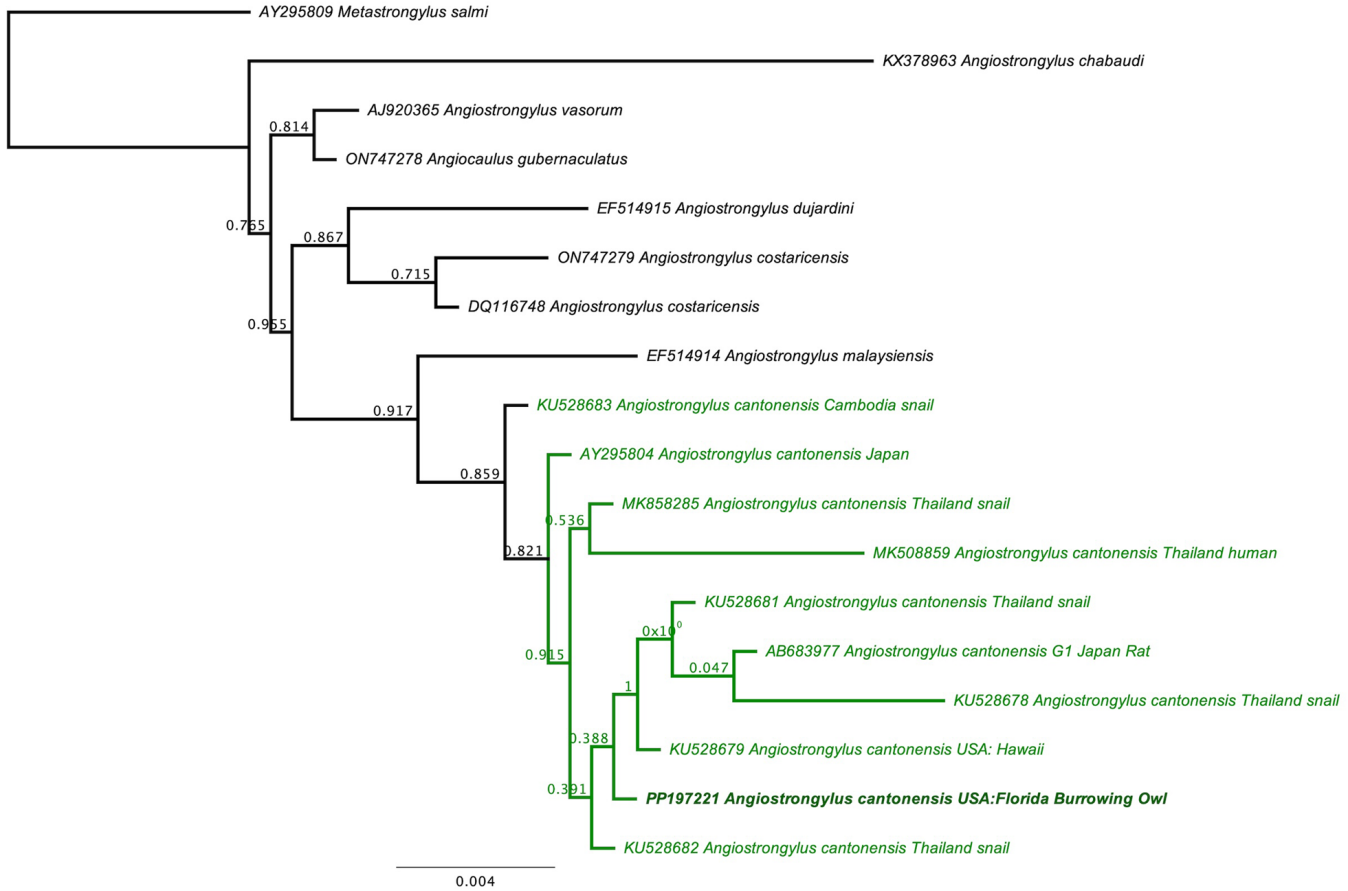
Phylogenetic relationship of an *Angiostrongylus cantonensis* 18S rRNA gene sequence (849 bp) from a burrowing owl (*Athene cunicularia*) from Florida with other *A. cantonensis* sequences and related taxa. *Angiostrongylus cantonensis* sequences are highlighted in green and our new sequence (GenBank PP197221) is in bold

A brain sample from owl A tested PCR positive for *A. cantonensis* DNA using the 18S rRNA gene sequence protocol. The 914 bp sequence was 99.9% similar to a lab strain of *A. cantonensis* from Japan (AY295804), followed by several other *Angiostrongylus* spp. (only 98.6–99% similarity); however, our sequence was 100% similar to five other *A. cantonensis* sequences in GenBank that were shorter (805–845 bp). This unique gene sequence was submitted to GenBank (accession number PP197221). Phylogenetically, our *A. cantonensis* sequence grouped with numerous *A. cantonensis* sequences from the USA (Hawaii), Thailand, Cambodia, and Japan (Fig. 4).

A fresh brain sample from owl B tested PCR negative for *A. cantonensis*, and no other nematodes, viruses, or protozoa were detected in the brain of either fledgling.

### Case 8 (W23-481A)

This juvenile owl was found on Marco Island on 26 May 2023, and was admitted to a rehabilitation facility. Clinical signs included obtunded mentation; respiratory distress; pale and dry mucous membranes; discolored (yellow) skin with petechiae over the body and wings; and malodor (described as resembling dog feces). Within 48 h following treatment initiation (antibiotics and vitamin K), clinical signs improved but neurologic signs progressed, and included head tilt and tremors. Owing to poor prognosis, the owl was euthanized using isoflurane gas inhalation, and the carcass was frozen prior to submission for necropsy.

Postmortem evaluation revealed that the owl was in good nutritional condition with adequate musculature and adipose stores. No gross lesions were evident. Histologically, numerous (~10 cross sections), widely scattered nematodes expanded the meninges over the cerebrum and cerebellum, and were within the cerebral neuroparenchyma. These nematodes were surrounded by severe inflammation, composed of variable proportions of multinucleated giant cells, histiocytes, lymphocytes, plasma cells, and eosinophilic granulocytes (Fig. 3C). Variable (mild to marked) lymphocytic and histiocytic inflammation extended to the meninges overlying the cervical spinal cord, which was multifocally expanded by heterophilic granulomas containing degenerated nematodes (Fig. 3D). Nematodes were ~100 μm diameter (cross sections), had a ~5-μm thick, smooth cuticle; coelomyarian–polymyarian musculature; a pseudocoelom containing prominent lateral chords and alae; and a digestive tract composed of uninucleated cells. The paraspinal ganglia contained a few small aggregates of lymphocytes.

Several degenerated nematode fragments primarily composed of large cuticles and intestines were observed in the squash preparation of the brain sample. The 66 k-kDa sequences of these nematodes were 100% similar to numerous existing *A. cantonensis* sequences. No avian influenza viruses were detected in a pooled oropharyngeal and cloacal swab sample, no viruses were isolated by cell culture, and no protozoans were detected by PCR test.

### Case 9 (W23-481C)

This juvenile owl was found alive, floating in a canal on Marco Island and was admitted to a rehabilitation facility on 30 June 2023. Physical evaluation revealed water-soaked feathers, and it was hypothermic and mentally obtunded. It subsequently was held in a warm incubator and administered supplementary oxygen. Within 30 min of initiation of supportive care, it started seizing and developed horizontal nystagmus, which failed to improve, and thus, the owl was euthanized using isoflurane gas inhalation. The carcass was stored frozen prior to submission for necropsy.

Postmortem evaluation revealed that the owl was in good nutritional condition with adequate musculature and adipose stores, and moderate to marked autolysis. There were no significant gross nor histologic lesions.

A brain sample tested positive using the 66-kDa PCR. The sequence was 100% similar to *A. cantonensis*. Ancillary testing failed to detect avian influenza virus in a pooled choanal and cloacal swab, or other viruses by culture, or protozoans by PCR.

### General findings for all cases

Among all owls examined histologically, systemic vascular congestion was consistently marked, and most had minimal to mild hepatic extramedullary hematopoiesis (except for W20-374B and C, and W21-269A) considered within normal limits for birds of the represented ages. The cloacal bursa was histologically evident in W20-374A, B, and C, W21-269A, and W23-481A, consistent with immature age, but may not have been grossly evident and thus not trimmed among all juvenile owls. Autolysis was moderate to marked in tissues from all owls, potentially masking some histologic lesion patterns.

## Discussion

We report the first cases of *A. cantonensis* infection in a free-ranging avian host in North America; the Florida burrowing owl. In the USA, the only previous avian report was in a captive African Pygmy falcon (*Polihierax semitorquatus*) in California. However, this case was not believed to have been acquired locally, but rather from the feeding of infected feeder geckos from Asia [52]. Worldwide, there have been few avian host species documented, with most cases being reported in Australia in free-ranging tawny frogmouths, captive cockatoos, and brolga (*Antigone rubicunda*) [7, 17, 53]. It is unknown if the lack of avian infections is owing to lack of neurologic testing of dead birds, or limited exposure, or low susceptibility, but experimentally, both domestic chickens and Japanese quail (*Coturnix japonica*) were resistant to infection [54].

Burrowing owls are a state threatened species in Florida, where they are facing increasing habitat loss, so populations have been pushed into urban environments. In general, burrowing owls have a broad diet, including: invertebrates, such as insects (e.g., beetles and roaches), spiders, and crayfish; herpetofauna, such as small snakes, lizards, and Cuban tree frogs (*Osteopilus septentrionalis*); birds; and rodents [55–57]. Burrows on developed properties offer the advantage of good lighting and irrigation, leading to increased availability of food sources such as frogs, lizards, and insects. Burrowing owls also commonly “decorate” their burrows with various man-made and natural items, including manure, which may attract insects and other potential *A. cantonensis* paratenic hosts to the burrow that owls may prey on [58]. Although data on possible paratenic hosts in Florida are limited, *A. cantonensis* has been reported in Cuban tree frogs, an invasive species in Florida [10]. This exotic parasite has become established in many regions of the southeastern USA owing to suitable climate, availability of intermediate gastropod hosts, and the presence of numerous mammalian hosts in which this nematode can achieve sexual maturity [2, 6, 20, 23, 26, 29]. However, the impact of this parasite on wildlife populations is unknown and requires additional research.

Angiostrongylosis is an important public health concern throughout its range, including the southern USA [6, 20, 59–61]. However, *A. cantonensis* has been listed as a “neglected pathogen” owing to lack of awareness among the general public, and potential under-reporting within the medical community [4]. In addition, this parasite is an important cause of neurologic disease in domestic dogs [16, 62–64]. Our focus of owl infections was on Marco Island, Florida, an island with high urban density and presence of domestic animals, so increasing knowledge for use by physicians and veterinarians in the region is important.

*Angiostrongylus cantonensis* is an increasingly important differential etiology of neurological disease in free-ranging wildlife and domestic species in the southeastern USA. Several of the known mammalian hosts are also susceptible to rabies virus-associated disease, so diagnostic evaluation and testing must be done using appropriate biosafety precautions. Currently, the primary method to diagnose this parasite is a “squash prep” examination of brain tissue before or after detection of the parasite in histologic sections [26]. In some cases (e.g., woodrat and opossums), large nematodes were grossly observed on the surface of the brain [25]. However, in many previously published cases, along with the burrowing owls in this study, parasites were not observed grossly at necropsy, and larvae were only detected on a squash prep or histologically [25, 52]. The latter is not always reliable as an indicator of infection, because histologic visualization of the nematodes is dependent on sections examined, and thus, parasites can easily be missed. Molecular testing can then be used to further assess and/or confirm parasite identification. Because birds of prey with neurologic signs are commonly admitted to rehabilitation centers and veterinary clinics, this parasite should now be considered along with other possible causes (e.g., trauma, West Nile virus, eastern equine encephalitis virus, highly pathogenic avian influenza virus, *Toxoplasma gondii*, and *Sarcocystis neurona*) [65–68]. In general, biologists, veterinarians, rehabilitators, wildlife managers, and pathologists should be aware of the parasite to ensure accurate detection and diagnosis in suspected cases. Antemortem diagnosis is difficult, as the clinical signs are nonspecific and eosinophilic pleocytosis may only be present in cerebrospinal fluid (CSF) for a limited time [24, 69, 70]. It is also possible to detect DNA of *A. cantonensis* in CSF if the sample is available, but in addition to the challenge and health risk of acquiring this sample in small wildlife species, a negative result does not rule out infection [20, 70, 71]. Thus, diagnosis is often not made until full postmortem evaluation (including histopathology and/or laboratory testing) is completed. Treatment options for infected aberrant hosts (e.g., dogs, nonhuman primates, and humans) with neurological signs generally are limited to supportive care, and sometimes high doses of glucocorticoids in attempt to control inflammation, analgesics to control pain, and anthelmintics to kill migrating larval stages (if suspected); however, killing large numbers of parasites quickly can cause more severe disease [72]. Therapeutic data for avian hosts are limited. To the author’s knowledge, there is only one study conducted on tawny frogmouth and yellow-tailed black cockatoo *(Calyptorhynchus funereus*), which, despite being treated with dexamethasone (0.7 mg/kg intramuscularly (IM) q12h or 4 mg/kg IM), did not show clinical improvement, and had to be euthanized [53].

The burrowing owls on the highly urbanized Marco Island have unique risks compared with owls that inhabit more natural grasslands. For example, urbanization carries a baseline risk of vehicular strikes, which can be increased for owls with neurological abnormalities, especially when flying at night. Concurrent work on other burrowing owl mortalities on Marco Island has revealed frequent high exposure rates to rodenticides, which can manifest with neurological signs similar to head trauma from vehicle strike or *A. cantonensis* infection (SCWDS, unpublished data). In fact, Cases 1 and 2 had multiorgan hemorrhage that could have resulted from trauma and/or rodenticide exposure, a finding that emphasizes the need for thorough diagnostic evaluation to accurately identify co-morbidities.

## Conclusions

*Angiostrongylus cantonensis* is widespread in the southeastern USA and is believed to be endemic (and probably emergent) throughout much of Florida [10, 20, 26, 29]. However, reports are sporadic so there may be important differences in prevalence or distribution among regions or habitats in the state. Rats are the definitive host for the parasite, and in urban areas they can reach very high densities. In particular, southern Florida has high rat densities in urban areas (Piersma, personal communication). Marco Island has a high density of homes, condominiums, hotels, restaurants, and other businesses, and a large percentage of them use some form of pest control for rats, often anticoagulant rodenticides. Thus, education campaigns for *A. cantonensis* should focus heavily on rodent control to dampen risk of transmission. However, given the health threat of anticoagulant rodenticides to owls, other wildlife, and domestic animals, control measures should balance rodent control and rodenticide risks, and there is an urgent need to investigate and develop alternative rodent control methods that do not rely on rodenticides.

## Data Availability

Sequence data that support the findings of this study have been deposited in GenBank (accession number PP197221) or are included in this published article.
